# *In ovo* Administration of Nucleosides Improved the Performance, Apparent Metabolizable Energy and Gut Development in Broiler Chickens

**DOI:** 10.3389/fvets.2020.583748

**Published:** 2020-12-11

**Authors:** Marappan Gopi, Villavan Manojkumar, Ashok Kumar Verma, Putan Singh, Jaydip Jaywant Rokade, Beulah V. Pearlin, Madheswaran Monika, Velusamy Madhupriya, Manimaran SaravanaKumar, Tamilselvan Tamilmani

**Affiliations:** ^1^Division of Avian Physiology and Reproduction, Central Avian Research Institute, Uttar Pradesh, India; ^2^Division of Animal Nutrition, Indian Veterinary Research Institute, Uttar Pradesh, India

**Keywords:** nucleosides, *in ovo*, gut development, performance, broiler

## Abstract

An *in ovo* study on the effect of the administration of a combination of nucleosides (25, 50, and 100 mg/egg) on hatchability, growth performance, energy metabolizability, and intestinal morphology in broilers was carried out. Four hundred eighty (480) fertile eggs were divided into four groups (in four replicates each having 30 eggs). On the 18th days of incubation of the eggs, candling was carried out and the fertile eggs were selected and given one of the four *in ovo* administrations. Group one served as control and was injected with phosphate-buffered saline (PBS). The other groups were given *in ovo* administration of nucleosides (25, 50, and 100 mg/egg) at 100 μl through the yolk sac route, and chicks of respective groups were hatched out. Among the experimental groups, the hatchability was comparable; however, the hatchability was affected in the group injected with a higher level of nucleosides at 100 mg/egg. The hatched out chicks from higher doses of nucleosides (50 and 100 mg) had higher body weight (BW) (*P* < 0.05) than the control. Higher energy metabolizability (%) was observed in nucleoside-injected groups. Plasma protein concentration was higher in groups administered with nucleosides (50 and 100 mg). Histologically, the intestinal villi length was maximum in 100 mg-injected group followed by 50 and 25 mg. Relative expression of homeobox (Cdx) in the jejunum was significantly (*P* < 0.05) upregulated in all the injected groups at 3, 7, and 14 days of age. Nucleoside-administered groups had better performance, energy metabolizability, and intestinal morphology. Among the experimental groups, the administration of nucleosides at 50 mg/egg resulted in higher growth performance, plasma protein, intestinal surface, and villi development in broiler chickens.

## Introduction

Genetic improvement paved the way for heavier birds with improved feed utilization efficiency under reduced rearing periods. As the productivity of birds increases, the demand of embryos for nutrients does changes. Early functions of the digestive tract are vital for chicken's growth and optimum muscle development. The small intestine undergoes both morphological and molecular changes during incubation. These changes are essential for the birds to adapt to rapid transition from yolk to physical nutrient sources. The changes were quite evident as the weight of the intestine reaches about 3.5% of embryonic weight at hatch from 1% at 17 days of incubation (1). From the 19th day, the yolk sac's internalization into the body cavity takes place and serves as source of energy following hatching until the exposure to feed (2–4).

In broilers of 35 days growing period, 37% of their life span is spent in incubation (21 days) at hatchery (5). Any interventions during incubation that promotes/accelerates growth would have a significant impact on post-hatch performance. The use of *in ovo* technique (administration of nutrients or vaccines) had resulted in beneficial effects during their post-hatch performance. Moreover, this prenatal feeding triggers the intestinal development by enhancing villi development and intestinal capacity to digest and absorb nutrients and provides a basis for muscle growth (6–8). Similarly, the *in ovo* administration of nanoparticles of calcium carbonate accelerated the bone development in broilers (9). For *in ovo* injection, with more area, the yolk sac route is an ideal site for administration (5). A recent study indicated that the use of prebiotic (galactooligosaccharides) through *in ovo* route mitigated the negative effects of heat stress in broiler chickens (10). It is quite evident that any interventions during embryonic stage has a positive effect of post-hatch performance in broiler chickens.

Nucleotides (low molecular weight compound) are the materials for nucleic acids. Nucleotides consist of a pentose sugar, nitrogenous base, and phosphate group, whereas nucleosides consist of all the above except phosphate group (11). Nucleotides play critical roles in many biological processes in the body. Rapidly proliferating tissues such as immune system and intestinal mucosal cells during stress and early growth periods require more amount of nucleotides which cannot be supplied merely by *de novo* synthesis. The salvage pathway, which harvests nucleobases from blood and diet, could support their demands (12). Nucleotides are involved in gastrointestinal tract and skeletal muscle development, and immune response (13).

The *in ovo* supplementation of nucleotides will play an important role in developing various systems, especially the gastrointestinal and immune systems. During the embryonic stage, the intestinal mucosal barrier and enzyme system is immature and hence the administered quantity will enter into the bird's body without any loss (14, 15). Since nucleotides are converted to nucleosides after absorption, this study was undertaken to assess the their effect following *in ovo* administration at different dosages on growth performance, energy metabolizability, plasma total protein, uric acid, villi length, and expression of gut development genes.

## Materials and Methods

### Ethical Approval

All experimental procedures involved in the study, such as the rearing of experimental birds and sampling, were approved by the institute animal ethics committee and members of CPCSEA nominees. The IAEC approval number is CARI/CPCSEA/2017/07.

### Birds and Sampling

A total of 480 fertile eggs of white commercial broiler chicken (CARIBRO Vishal) were incubated in an incubator. All the eggs were incubated at normal incubation temperature (99.5°F−99.75°F) and relative humidity (50–60%) for initial 17 days. After 17 days of incubation, the eggs were divided into four treatments, each consisting of 120 eggs for i*n ovo* injection. Each group was consisted of four replicates having 30 eggs in each. On the 18th day, the eggs were candled, and the fertile eggs were reweighed and transferred to the laminar airflow for *in ovo* administration of nucleosides. Out of the four groups, eggs in the first group served as control which was injected with phosphate-buffered saline (PBS). The other three were treatment groups and were given *in ovo a*dministration of nucleosides (25, 50, and 100 mg/egg) at the rate of 100 μl through the chorioallantoic membrane using tuberculin needles and deposited into the yolk sac as per the protocol of Bhanja et al. (16). The doses were based on our earlier *in vivo* experiments (17). Commercial nucleosides (adenosine, guanosine, cytosine, and uridine−100% purity) were used in the study (HiMedia India Pvt. Ltd., Mumbai, India), mixed in equal proportion, and suspended in autoclaved PBS. Immediately after injection, the site was sealed with sterile paraffin. All the eggs were transferred to a hatcher, and the chicks were hatched out.

### Hatchability Percentage and Experiment Diets

The hatchability percentage was calculated on fertile egg basis, and break out analyses was performed to study the causes for embryonic mortality. Following hatching, the chicks from respective groups were pulled out, wing banded, weighed, and grouped into respective groups and labeled. The hatched out chicks of respective groups (five replicates) were reared with common broiler feed for 42 days. The birds were fed with pre-starter (1–14 days), starter (15–28 days), and finisher (29–42 days) diets as per the nutritional recommendations of the Indian Council of Agricultural Research (18). The physical and chemical composition of the experimental diet is presented in Table 1.

**Table 1:** Feed ingredients and chemical composition of experimental diet (as-fed basis).

**Ingredients (%)**	**Pre-starter**	**Starter**	**Finisher**
Corn	546.0	542.0	576.2
Soybean meal (48% CP)	395.8	378.0	325.8
Rice bran oil	21.20	42.40	58.60
Calcite	15.40	15.20	17.30
Di-calcium phosphate	9.00	9.50	11.00
Salt	1.80	1.80	1.80
L-Lysine	3.00	1.50	1.70
DL-Methionine	3.00	2.80	2.70
Phytase	0.15	0.15	0.15
Mineral premix^a^	0.15	0.15	0.15
Vitamin premix^b^	0.14	0.14	0.14
Coccidiostat^c^	0.10	0.10	0.10
Toxin binder^d^	0.50	0.50	0.50
**ANALYZED VALUES**			
Crude protein	226.5	216.5	197.0
Metabolizable energy (MJ/kg)	12.55	13.08	13.60
Calcium	9.60	9.50	9.00
Available phosphorus^*^	4.50	4.60	4.60
Lysine^*^	14.20	12.50	11.40
Methionine^*^	6.20	5.90	5.50

* *Calculated values*.

a *Mineral premix composition: 91 mg manganese, 91 mg zinc, 85 mg iron, 1.82 mg iodine, 30.24 mg copper, and 0.365 mg cobalt/kg*.

b *Vitamin premix composition: 16,500 IU retinol, 3,200 IU cholecalciferol, 2 mg menadione, 5 mg thiamine, 13 mg riboflavin, 8 mg pyridoxine, 320 mg niacin, 0.05 mg cyanocobalamin, 95 mg DL-α-tocopherol, 27.5 mg calcium D pantothenate, 14 mg folic acid/kg*.

c *Coccidiostat supplied 125 mg Dinitro-ortho-toluamide/kg*.

d *Toxin binder composition: blend of organic acids, hydrated sodium calcium aluminum silicate, mannan oligosaccharides, and oxine copper (Check-O-Tox, Zoetis, India)*.

### Energy Metabolizability and Production Performance

A metabolism trial was conducted after 14 days of age, with 4 days of collection period and energy metabolizability was studied. The birds were kept individually in metabolic cages. The feed was offered to the birds in the cages in a separate feeder. Simultaneously, the residue was collected next day. The excreta were collected daily at the morning (10 a.m.), weighed, and stored appropriately for further analyses. Samples of experimental diets together with droppings were chemically analyzed for gross energy (GE) estimation using an adiabatic bomb calorimeter. The apparent metabolizable energy (AME) was determined by utilizing the balance data and the GE content of diets and droppings (19). The replicate feed consumption at different phases (14th, 28th, and 42nd day of age) and body weight (BW) at similar stages were recorded.

$$\begin{array}{l} \text{AME }\left(\text{kcal}/\text{kg}\right)=\;\frac{\left(\text{FC }\times \text{ G}{\text{E}}_{\text{f}}\right)-\left({\text{E}}_{\text{w }}\times \text{ G}{\text{E}}_{\text{e}}\right)}{\text{FC}} \end{array}$$

Where,

FC, feed consumption (g/bird/day)

*E*_w_, dried excreta weight (g/bird/day)

GE_f_, gross energy of feed (kcal/kg)

GE_e_, gross energy of excreta (kcal/kg).

### Plasma Total Protein and Uric Acid Content

On 0, 3, 7, 14, 21, and 42 days of age, 10 birds (one male and one female per replicate) from each group were sacrificed in the morning before feeding, and blood samples were collected from the jugular vein in anticoagulant-coated tubes. The tubes were centrifuged at 2,500 rpm for 20 min to separate the plasma, and it was stored at −20°C until analysis. Plasma total protein and uric acid contents were quantified using Coral Clinical Systems, Tulip Diagnostics (P) Ltd., Goa. Plasma total protein and uric acid concentration were estimated by biuret and Uricase/PAP method, respectively.

### Intestinal Gross and Histomorphology

Ten birds from each group were randomly selected and sacrificed (days 0, 7, and 14) by cervical dislocation and the whole intestinal segment was removed from the birds. Both the weight and length were measured and expressed in percent BW and centimeter per kilogram BW, respectively. The midpoint from the pancreatic loop to the Meckel's diverticulum about 1 cm of the jejunum part was excised, flushed with normal saline to remove the contents, and fixed in 10% neutral-buffered formal saline for histological analysis. For each sample, both longitudinal and cross sectional segments were made and analyzed under light microscope. Intestinal villi length was measured from tip of the villi to the villus-crypt junction using Zeiss microscope blue core software, and for each sample, six measurements were recorded.

### Relative Expression of Intestinal Development Gene (Cdx)

The relative expression and quantification of Cdx gene in the jejunum was quantified by real-time PCR. The jejunum samples (*N* = 40; 10 per treatment) were collected in RNA*later* at 3, 7, and 14 days of age and stored at −20°C until further processing. The total RNA was isolated from the jejunum by TRIzol (Invitrogen, USA) extraction method. The quality of isolated RNAs was assessed by the absorbance ratio at 260–280 nm using a microvolume spectrophotometer (NanoDrop®, Thermo Scientific Fischer, USA). The RNAs having a ratio value of 1.8–2 were taken for further processing. The first strand cDNA were synthesized using RevertAid cDNA Synthesis Kit (MBI Fermentas, USA). PCR and qPCR were carried out in thermal cycler and real-time cycler (Bio-Rad Laboratories, USA) using standard conditions. All the samples were run in triplicate with non-template control (NTC) included in each PCR reaction to check DNA contamination. Oligo-nucleotide sequence of gene primers forward and reverse are provided in Table 2. GAPDH was used as a reference gene.

**Table 2:** Oligonucleotide sequences for Cdx gene expression studies.

**Gene**	**Primer sequence**	**Annealing temperature**	**Accession no.**
Cdx	F-CTCGGACTTCGCCAGCTACC	56.0°C	AB046532
	R-TGCGCCTCATCCATTCGTAC		
GAPDH	F-GTGTGCCAACCCCCAATGTCTCT	58.2°C	K01458
	R-GCAGCAGCCTTCACTACCCTCT		

### Statistical Analysis

Data collected on various parameters were subjected to analysis of variance using Statistical Package for Social Sciences version 16.0. The observations on hatchability, body weight, and feed intake were subjected to single-factor analysis. The observations on plasma total protein, plasma uric acid, gross intestinal morphology, and jejunal villi length were subjected to two-factor analysis to interpret the effect of treatment, duration, and its interaction. The means were compared for significance using Tukey's range test.

## Results

### Hatchability

The effect of *in ovo* nucleoside administration on egg weight during incubation, the percentage of hatchability, and embryonic mortality is presented in Table 3. There was a reduction in egg weight during incubation on the 18th day compared to day 0. The hatchability was comparable among the injected groups; however, the hatchability was affected in groups injected with a higher level of nucleosides at 100 mg/egg. The unhatched eggs were broken out, and the cause of embryonic mortality was found out. The results of egg break analysis of unhatched eggs did not show any group-specific lesions, and the pattern of mortalities were uniform among the groups.

**Table 3:** Effect of *in ovo* nucleoside administration on the hatchability (%) and embryonic mortality (%) in broiler chickens.

**Group**	**Egg weight (g)**		**Hatchability (%)**	**Embryonic mortality (%)**			
	**Day 1**	**Day 18**		**Dead in shell**	**Infected**	**Dead after injection**	**Live pipping**
Control	60.78	52.35	75.97	12.00	3.00	7.92	1.11
25 mg	60.20	53.48	78.55	10.89	3.22	7.34	0.00
50 mg	59.46	53.43	78.33	11.54	3.45	8.23	1.51
100 mg	59.56	53.48	74.76	8.78	3.33	10.69	2.44

### Energy Metabolizability and Production Performance

No significant difference among the groups was observed at fortnight and overall feed intake, except during the fourth week of experiment (Table 4). At the fourth week, the birds under higher administered dose (100 mg) had consumed more (*P* < 0.05) feed than the other three groups. The birds hatched out from the groups that are injected with higher levels of nucleosides (50 and 100 mg) had higher BW (*P* < 0.05) than the control (Table 5). The birds injected with lower dose (25 mg) exhibited intermediate response. The energy metabolizability (%) among the different groups showed a significant (*P* < 0.01) difference at 14 days of age (Table 6). Higher energy metabolizability (%) was observed in the *in ovo*-injected birds (25, 50, and 100 mg) when compared to the control group (83.79%, 86.65%, and 84.46 vs. 77.13%).

**Table 4:** Effect of *in ovo* nucleoside administration on the feed consumption (g/bird) and energy metabolizability (%) in broiler chickens.

**Group**	**2nd week**	**4th week**	**6th week**	**Overall**	**Energy metabolizability (%)**
Control	539	1,038^c^	2,053	3,630	77.13^b^
25 mg	537	1,093^b^	2,026	3,656	83.79^a^
50 mg	557	1,096^b^	2,037	3,690	86.65^a^
100 mg	573	1,143^a^	2,101	3,717	84.46^a^
SEM	1.24	4.70	10.88	16.87	0.02
*P*-value	0.241	0.031	0.094	0.134	0.001

*Means within column bearing different superscripts differ significantly (P < 0.05)*.

**Table 5:** Effect of *in ovo* nucleoside administration on the body weight (g) in broiler chickens.

**Group**	**Hatch weight**	**2nd week**	**4th week**	**6th week**
Control	46.2	449.1^b^	1,091.7^b^	2,026.3^b^
25 mg	46.3	447.1^b^	1,134.8^ab^	2,072.9^ab^
50 mg	46.9	463.8^a^	1,188.5^a^	2,123.5^a^
100 mg	48.41	477.6^a^	1,189.9^a^	2,162.4^a^
SEM	0.36	3.25	12.19	14.03
*P*-value	0.232	0.036	0.043	0.031

*Means within column bearing different superscripts differ significantly (P < 0.05)*.

**Table 6:** Effect of *in ovo* nucleoside administration on the plasma total protein (g/dl) concentration in broiler chickens.

**Group**	**Period (days)**						**Group mean**	***P*****-value**			**SEM**
	**0**	**3**	**7**	**14**	**21**	**42**		***G***	***P***	***G* × *P***	
Control	3.36^h^	3.66^gh^	5.14^e^	5.90^b^	5.72^c^	5.18^e^	4.83^z^	0.033	0.001	0.016	0.17
25 mg	4.02^fg^	4.30^f^	5.86^bc^	5.30^de^	6.02^b^	6.35^a^	5.14^xy^				
50 mg	3.55^gh^	4.49^f^	5.74^c^	5.75^c^	6.11^ab^	6.43^a^	5.35^x^				
100 mg	3.74^gh^	4.28^f^	5.47^d^	5.39^d^	6.14^ab^	6.64^a^	5.28^x^				
Period mean	3.67^C^	4.18^C^	5.55^AB^	5.59^AB^	6.00^A^	6.15^A^					

*Means bearing different superscript within column (x–z), row (A–C), and interaction (a–h) differ significantly (P < 0.05)*.

*G × P, group × period interaction*.

### Plasma Total Protein and Uric Acid

The plasma concentration of total protein showed significant difference (*P* < 0.05) due to treatment, period, and its interaction (Table 6). The results revealed higher protein concentration in groups administered with higher nucleosides (50 and 100 mg) when compared to the control group. Similarly, the protein content showed age-dependent linearity and reached a higher concentration at 42 days of age. The interaction effect revealed a lower protein concentration at initial stages (days 0 and 3), and then, the values showed a steady increase with higher levels in supplemented groups than the control. The result for the concentration of plasma uric acid (mg/dl) is furnished in Table 7. Group mean showed no significant difference (*P* > 0.05), while the period mean exhibited a significant (*P* < 0.05) reduction in their concentration as the age increases. The interaction did not reveal any treatment × period association in the levels of serum uric acid.

**Table 7:** Effect of *in ovo* nucleoside administration on the plasma uric acid (mg/dl) content in broiler chickens.

**Group**	**Period (days)**						**Group mean**	***P*****-value**			**SEM**
	**0**	**3**	**7**	**14**	**21**	**42**		***G***	***P***	***G* × *P***	
Control	5.89	5.59	5.44	5.32	5.41	5.17	5.47	0.511	0.001	0.316	0.325
25 mg	6.07	5.95	5.56	5.46	5.39	5.29	5.62				
50 mg	6.18	5.98	5.68	5.48	5.23	5.16	5.62				
100 mg	6.22	6.02	5.76	5.55	5.47	5.24	5.71				
Period mean	6.09^A^	5.89^A^	5.61^B^	5.45^BC^	5.38^BC^	5.22^C^					

*Means bearing different superscript differ significantly (P < 0.01)*.

*G × P, group × period interaction*.

### Intestinal Morphology and Development

The intestinal length and weight were comparable (*P* > 0.05) among the groups, but their weight showed period mean significant difference (*P* < 0.01) (Table 8). The results showed that intestinal length at days 0 and 7 differed significantly (*P* < 0.01) from day 14 and intestinal weight at day 0 significantly differed (*P* < 0.01) from other periods. Reduction in the intestinal length has been observed on day 14 when compared to other periods. Among all periods, day 7 had the highest intestinal length and weight. The least length and weight were noticed on day 14 and day 0, respectively. Interaction study of intestinal morphology revealed that the control group had higher intestinal length and weight. The least intestinal length was observed in the control group at day 14 and the least weight was observed in the 50-mg group at day 0. Other groups exhibited an intermediate response. All the groups showed a reduction in intestinal length and weight per unit from day 7–14.

**Table 8:** Effect of *in ovo* nucleoside administration on the intestinal gross morphology in broiler chickens.

**Group**	**Period (days)**			**Group mean**	***P*** **value**			**SEM**
	**0**	**7**	**14**		***G***	***P***	***G* × *P***	
**Intestinal length (cm/kg body weight)**								
Control	815.56^abc^	910.47^a^	529.92^bcd^	751.98	0.446	0.001	0.005	28.991
25 mg	902.55^a^	963.75^a^	488.27^cd^	784.86				
50 mg	822.03^abc^	789.39^abcd^	487.74^cd^	699.72				
100 mg	808.97^abcd^	891.76^a^	634.77^abcd^	778.50				
Period mean	837.28^A^	888.84^A^	535.18^B^					
**Intestinal weight (% body weight)**								
Control	38.52^c^	149.25^a^	139.01^a^	108.93	0.286	0.001	0.001	7.267
25 mg	52.09^bc^	153.44^a^	128.39^a^	111.31				
50 mg	39.96^c^	137.33^a^	126.89^a^	101.39				
100 mg	34.01^c^	133.79^a^	131.89^a^	99.90				
Period mean	41.15^B^	143.45^A^	131.55^A^					

*Means bearing different superscript within row (A–B) and interaction (a–d) differ significantly (P < 0.01)*.

*G × P, group × period interaction*.

**Figure 1:**
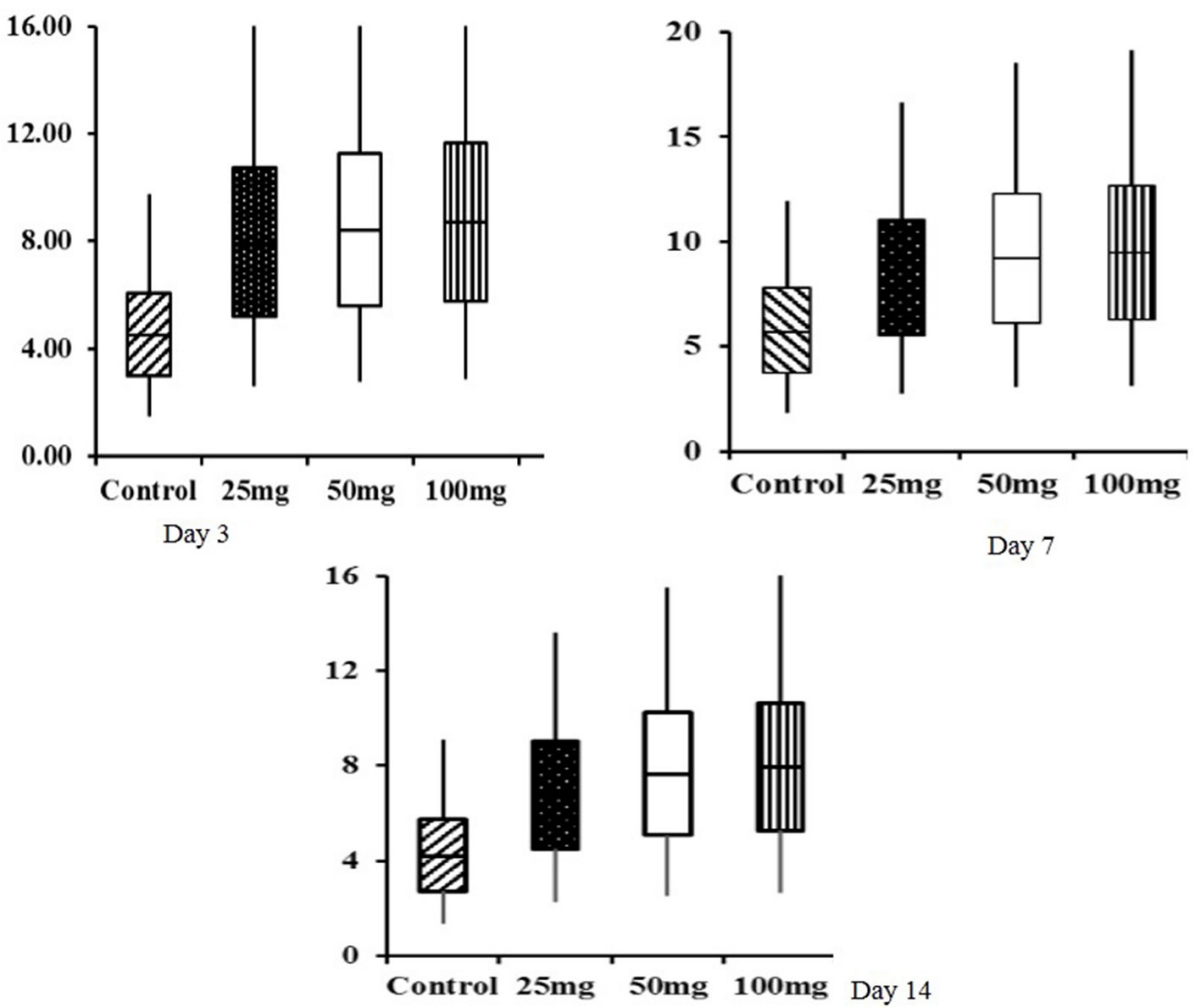
Box plot analysis of Cdx gene in jejunum tissue of *in ovo* injected nucleosides broiler chickens. Box plots, box shows the lower quartile, median (dark line), mean (+ symbol), and upper quartile values and the whisker's show the range of relative expression of Cdx gene in jejunum tissue at 3, 7, and 14 days of age.

Histomorphological results showed that both group mean and period mean were significantly different (*P* < 0.01). The highest intestinal villi length was observed in the 100-mg-dose-injected group, followed by 50 and 25 mg (Table 9). The period means exhibited a significant increase in the pattern of villi length up to 42 days. Interaction study revealed that the 100-mg group had the highest villi length on day 42, and the lowest length was observed in the control group at day 0. The relative expression of homeobox (Cdx) gene in the jejunum showed significant (*P* < 0.05) upregulation in the injected groups at 3, 7, and 14 days of age (Figure 1). The magnitude of expression is higher during 7 days of age after hatch, whereas the expression is lower at days 3 and 14.

**Table 9:** Effect of *in ovo* nucleoside administration on the jejunal villi length (μm) in broiler chickens.

**Group**	**Period (days)**					**Group mean**	***P*****-value**			**SEM**
	**0**	**3**	**7**	**14**	**42**		***G***	***P***	***G* × *P***	
Control	1,255.53^j^	1,658.64^i^	2,245.79^g^	2,849.17^ef^	5,326.72^c^	2,667.17^z^	0.001	0.001	0.001	19.80
25 mg	1,340.87^j^	1,839.45^i^	2,374.16^g^	3,074.81^de^	5,653.19^b^	2,856.50^y^				
50 mg	1,419.50^j^	1,894.52^hi^	2,749.47^f^	3,174.10^d^	5,880.21^ab^	3,023.56^x^				
100 mg	1,408.64^j^	2,119.41^gh^	2,918.21^e^	3,248.62^d^	5,917.50^a^	3,122.48^x^				
Period mean	1,356.14^E^	1,878.01^D^	2,571.91^C^	3,086.68^B^	5,694.41^A^					

*Means bearing different superscript within column (x–z), row (A–E), and interaction (a–k) differ significantly (P < 0.01)*.

*G × P, group × period interaction*.

## Discussion

### Hatchability (%)

The egg weights were comparable among the groups at both during the day of setting and after 18 days of incubation. About 10–12% of embryonic mortality was encountered after *in ovo* injection across the injected groups. Among the experimental groups, better hatchability was observed in 25 and 50-mg group. It indicated that the administration of nucleosides had an impact in the hatchability percentage of eggs. The hatchability percentage was negatively related to the concentration of nucleosides. Eggs with lower concentration at 25 mg/egg showed better hatchability than other eggs which had higher concentration of nucleosides. The hatchability percentage reduction might be due to alteration in the osmolarity inside the egg as the nucleosides were administered as suspension. The nucleosides are sparingly soluble in aqueous solvents, and the administration of nucleosides as a solution instead of suspension would have resulted in better hatchability percentage following *in ovo* administration. Further studies could be directed toward improving the solubility of these nucleosides by manipulating the pH of the solvents which could result in better hatchability.

### Growth Performance and Energy Metabolizability

There was a non-significant increase in feed intake of T3 group birds observed in all other three phases (pre-starter, finisher, and overall growth phase). Post-hatch feeding of nucleotides at 0.04, 0.05, 0.06, and 0.07% resulted in significantly higher feed consumption than the control group (20). Hatch weight did not show any significant difference among the groups, which indicated that the administration of nucleosides at the 18th day of incubation did not influence the weight at hatch. Post-hatch bi-weekly BW measurements revealed significant difference (*P* < 0.05) at all the growth phases and overall BW. Supplementation of 0.05% of commercial nucleotide products (Nucleoforce, containing 26.4% of balanced total nucleotides) in broiler diets resulted a significant increase in BW from 0 to 21 days of age (21). Similarly, in swine, supplementation of 4.0% of yeast extract product (NuPro^TM^ containing 7% of total nucleic acids) increased the BW (22). Incorporation of 2% nucleotide and other related compounds (NuPro) increased BW in broiler chickens when compared to control (23). This increase has been attributed to an increase in the digestibility and absorption of nutrients by the pancreatic and brush border digestive enzymes activity. A similar effect was also observed in the present study. The results further justified our earlier findings, where the dietary supplementation of nucleosides at 0.1% resulted in significantly higher BW and gain in broilers. Energy metabolizability results revealed that all the nucleoside-injected groups have higher energy metabolizability than other groups. It indicated that the administration of nucleosides had improved energy metabolizability in broilers. It might be due to the intestine's rapid cell turnover, which increases enzyme activity and energy metabolizability. A study carried out in broilers with supplementation of 2% nucleotides, NuPro, resulted in higher nutrient digestibility (23).

### Plasma Concentration of Total Protein and Uric Acid

There was an increasing pattern of concentration of total protein, albumin, and globulin observed up to 42 days. These results revealed that nucleoside-injected groups showed a higher protein concentration than the control group in all the periods. Intraperitoneal administration of nucleoside–nucleotide mixture increased total protein concentration in mice (24). This indicates that nucleotides are essential to increase body protein turnover rate, specific protein synthesis, and enterocyte proliferation. Nucleotides facilitate protein formation through messenger RNA synthesis (25). An age-related inverse relation was observed for uric acid due to the degradation of purine bases (26). The administration of nucleosides elevated the concentration of uric acid except at day 42. The higher values in the supplemented groups might be due to higher breakdown of nucleosides, which are then metabolized and excreted as uric acid.

### Intestinal Morphology and Development Studies

Intestinal length and weight per unit weight were reduced (*P* < 0.01) as the bird's age increases from 7 to 14 days. This is due to an increase in the proportion of BW higher than the length and weight of the intestine from day 7–14. An interaction study revealed that up to day 7, a lower dose-injected group (25 mg) had the highest intestinal length and weight among all groups. Nucleotides appear to stimulate the development of the intestinal lining and intestinal enzyme concentrations in animals. They also have beneficial effects on recovery from intestinal injuries caused by malnutrition or chronic diarrhea (27, 28). This positive effect on the gastrointestinal tract development might be due to enhanced DNA and RNA synthesis because of increased nucleotide pools following intake. This increased DNA and RNA synthesis enhances growth and differentiation of the enterocytes after injuries or malnutrition. Therefore, the administration of nucleosides, which can be converted to nucleotides, may help optimize tissue function in the gastrointestinal tract and stimulate the activity of brush border enzymes. These entire factors may be contributing in increasing intestinal weight.

The intestinal villi length results showed that nucleoside-administered groups were significantly different (*P* < 0.01) from other groups that were non-injected. In all the periods of measurements, the intestinal villi's length was increased in nucleoside-injected groups of broilers. The administration of nucleosides plays an essential role in developing the villi length in the small intestine of broilers. The increased villi length in all nucleoside-administered groups might be due to increased Cdx gene expression, which is the indicator of intestine development. Thus, the mucosal villi's rapid development allows chicks to utilize nutrients more efficiently in their early life and improve growth performance (29). The intestinal epithelium is a rapidly proliferating tissue with a high cell turnover rate, and dietary nucleotides are reported to play a role in the growth and differentiation of the gastrointestinal tract (30). Uauy et al. (27) observed an increased tissue protein, DNA content, as well as the activities of disaccharidase in the intestine of weanling rats fed with 0.8% *w*/*w* dietary nucleotide than control. Jung and Batal (23) also reported that birds fed on a diet supplemented with 0.25% torula yeast RNA and 2% NuPro, a commercial nucleotide product, had significantly (*P* < 0.05) higher villus heights as compared with the birds fed on the control.

Early weaning in piglets is the most stressful condition which adversely affects the intestine. The consistent occurrence of piglet diarrhea is the most common problem with early weaned piglets. Under this condition, the diet supplemented with 0.1% or 0.2% commercial nucleotide-rich yeast extract product (containing 25% nucleotides) had higher villus heights than the control group (31, 32). As nucleosides are the preferred form for absorption by enterocytes, nucleosides were administered in this study. All these effects were due to the administration of nucleosides, which were converted to nucleotides after absorption. Nucleosides also increase the villi length of the small intestine through rapid cell turnover.

The box-plot analysis of Cdx gene expression revealed upregulation in all the three doses of *in ovo* injection compared to both the controls. Intraperitoneal administration of nucleoside–nucleotide mixture increased small intestinal RNA levels in mice, which indicated an increased proliferation of enterocytes compared to the control group (24). Supplementation of nucleotides provides benefits to enterocyte function during normal periods of growth and development characterized by high demand for DNA and RNA synthesis (33).

## Conclusion

From the experiment, it could be concluded that nucleoside administration at the rate of 50 mg/egg resulted in higher growth performance, intestine surface, and villi development in broiler chickens. Further improvement can be made to overcome the reduction in hatchability percentage by improving the solubility of nucleosides.

## Data Availability Statement

The raw data supporting the conclusions of this article will be made available by the authors, without undue reservation.

## Ethics Statement

The animal study was reviewed and approved by Chairman, Institute Animal Ethics Committee, CARI. All experimental procedures involved in the study, such as the rearing of experimental birds and sampling, were approved by the institute animal ethics committee and members of CPCSEA nominees. The IAEC approval number is CARI/CPCSEA/2017/07.

## Author Contributions

MG, JR, and AV: designed the experimental design and analyzed the collected data. PS and VMad: prepared the manuscript. VMan, MM, and MS: carried out the *in ovo* administration, biological experiment, and performed sample collection. BP and TT: carried out the laboratory analysis of biological samples. All authors read and approved the manuscript.

## Conflict of Interest

The authors declare that the research was conducted in the absence of any commercial or financial relationships that could be construed as a potential conflict of interest.
